# Microglia Dynamics and Interactions with Motoneurons Axotomized After Nerve Injuries Revealed By Two-Photon Imaging

**DOI:** 10.1038/s41598-020-65363-9

**Published:** 2020-05-26

**Authors:** Travis M. Rotterman, Francisco J. Alvarez

**Affiliations:** 1 Department of Physiology, Emory University, Atlanta, GA 30322 United States of America; 2 School of Biological Sciences, Georgia Tech, Atlanta, GA 30318 United States of America

**Keywords:** Microglia, Neuroimmunology

## Abstract

The significance of activated microglia around motoneurons axotomized after nerve injuries has been intensely debated. In particular, whether microglia become phagocytic is controversial. To resolve these issues we directly observed microglia behaviors with two-photon microscopy in *ex vivo* spinal cord slices from CX3CR1-GFP mice complemented with confocal analyses of CD68 protein. Axotomized motoneurons were retrogradely-labeled from muscle before nerve injuries. Microglia behaviors close to axotomized motoneurons greatly differ from those within uninjured motor pools. They develop a phagocytic phenotype as early as 3 days after injury, characterized by frequent phagocytic cups, high phagosome content and CD68 upregulation. Interactions between microglia and motoneurons changed with time after axotomy. Microglia first extend processes that end in phagocytic cups at the motoneuron surface, then they closely attach to the motoneuron while extending filopodia over the cell body. Confocal 3D analyses revealed increased microglia coverage of the motoneuron cell body surface with time after injury and the presence of CD68 granules in microglia surfaces opposed to motoneurons. Some microglia formed macroclusters associated with dying motoneurons. Microglia in these clusters display the highest CD68 expression and associate with cytotoxic T-cells. These observations are discussed in relation to current theories on microglia function around axotomized motoneurons.

## Introduction

The modern era of microglia research was jump-started by the report of Blizinger and Kreutzberg in 1968^1^ describing the microglia reaction around facial motoneurons following nerve injury. However, despite the many theories proposed over the years, the function of microglia surrounding axotomized motoneurons is not yet settled. One new approach to gain new insights into this problem would be to directly visualize the interactions between microglia and axotomized motoneurons in real time using two-photon microscopy.

Blizinger and Kreutzberg first showed that after injury to the facial nerve, resident microglia in the facial nucleus proliferate and migrate towards axotomized motoneurons, surround the cell bodies, and interpose themselves between the motoneuron and synaptic boutons. These observations were made with electron microscopy and interpreted as “lifting” synapses in a phenomenon later coined as “synaptic stripping”^2–4^. Microglia attachment to the cell bodies and “synaptic stripping” were thereafter described on every type of motoneuron analyzed after axon injury^5–13^, as well as in other central and peripheral neurons disconnected from their targets by either axotomy or lesioning their axonal targets^14–18^. Whether synaptic stripping involves synapse phagocytosis or degradation requiring microglia activation has remained an unsolved and controversial question^19–21^.

Another proposed function for microglia interactions with axotomized motoneurons is neuroprotection. Early studies carried out in the hypoglossal nucleus found no evidence of excessive motoneuron cell death when microglia proliferation was inhibited^22^, but later studies using genetic manipulations to reduce the accumulation of activated microglia around facial motoneuron cell bodies found decreased survival rates after axotomy^23^. Neuroprotection is sometimes linked to synaptic stripping. Transient removal of synapses on motoneurons after axotomy affects predominantly excitatory synapses^12,24–27^ and this lead to the proposal that this process protects motoneurons from excitotoxicity by altering the E/I balance^28,29^. However, it is unclear whether microglia are directly involved in removing excitatory synapses from the motoneuron surface, with the exception of proprioceptive synapses originated in sensory axons also injured peripherally^30^. Moreover, the finding that the potassium-chloride cotransporter 2 (KCC2) is downregulated in axotomized motoneurons necessarily changes the way we interpret inhibitory synaptic function over regenerating motoneurons^31^.

An alternative neuroprotection mechanism involves microglia interactions with astrocytes and the adaptive immune system. In one model, infiltrated CD4 T-cells displaying a Th2 profile induce microglia to release interleukin (IL)-10 that primes astrocytes for exerting neuroprotection over facial motoneurons^32^. However, T-cell infiltration displays strong differences dependent on species, strain/genetic background, and injury type^30,33,34^. In a different model, astrocytes exert neuroprotection of axotomized facial motoneurons by upregulating the signal transducer and activator of transcription-3 (STAT3) dependent on IL-6 released from microglia^35^. Adult motoneuron survival after axotomy shows differences among species and type of injury^36–38^, despite all inducing similar microgliosis around axotomized motoneuron cell bodies^39^. The significance of direct microglia-motoneuron interactions is not well-understood.

It is clear that the functional significance of microglia surrounding axotomized motoneurons deserves investigation with new methods. An important step forward would be to directly observe microglia-motoneuron interactions with time-lapse microscopy. Microglia motility around axotomized motoneurons has only been analyzed in one early study using infrared gradient contrast microscopy in combination with video contrast enhancement and time-lapse recording^40^, but the resolution of this technique pales in comparison with the capabilities of modern two-photon microscopy combined with genetically tagged microglia, such as those in CX3CR1-GFP mice^41,42^. Unfortunately, imaging microglia in the spinal cord *in vivo* using CX3CR1-GFP mice has been limited to the study of interactions of microglia with white matter axons close to the spinal cord surface after crush injury to dorsal columns, experimental autoimmune encephalitis, amyotrophic lateral sclerosis, or spinal cord injury ^41,43–48^. Imaging of adult microglia interactions with neurons and synapses in the grey matter *in vivo* has been notoriously difficult because the surrounding myelinated white matter present a formidable optical barrier that diminishes resolution and sensitivity^49^. In addition, the spinal cord ventral horn is particularly difficult for surgical access and imaging^49^.

To investigate microglia dynamics around spinal motoneurons after axotomy we adapted for two-photon imaging an *ex vivo* adult spinal cord slice preparation first developed for *in vitro* electrophysiology^50^. This resulted in a significant improvement in resolution and sensitivity when imaging CX3CR1-GFP microglia. Another advantage of the slice preparation is that after unilateral nerve injury, comparison of the experimental side (ipsilateral to the injury) with the control side (contralateral to the injury), can be accomplished easily and rapidly by just moving the stage. Using this preparation, we describe for the first time dynamic interactions between microglia and motoneurons and how they change with time after nerve injury.

## Results

### Spinal cord slice preparation validation

**Figure 1:**
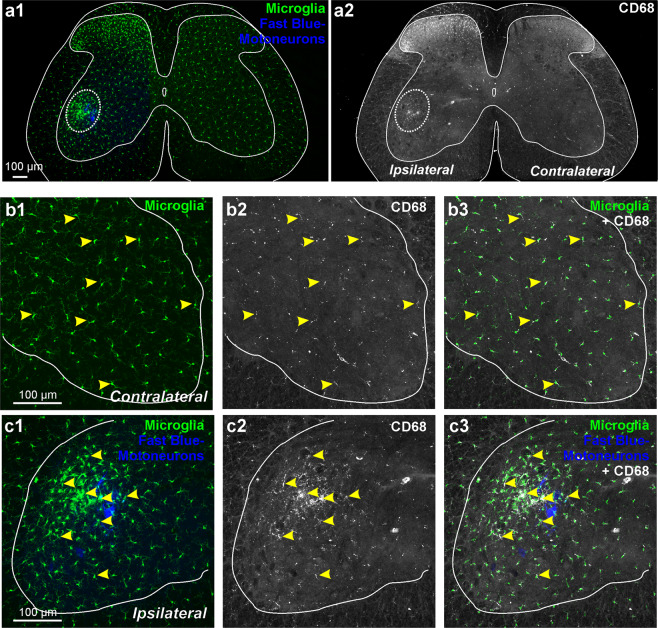
CD68 in mouse spinal microglia 14 days after injury. (**a1**) Low magnification confocal image of CX3CR1-GFP microglia in the spinal cord of an animal with an unilateral sciatic nerve lesion. Motoneurons ipsilateral to a sciatic nerve injury were retrogradely labeled with Fast Blue (FB). Microglia show increased numbers in the superficial laminae of the dorsal horn ipsilateral to the injury and around axotomized motoneurons in the ventral horn (dashed ellipse). (**a2)** CD68 immunoreactivity in the same section as in a1. CD68 is upregulated in dorsal and ventral regions showing microgliosis. (**b**) Higher magnification confocal image of the control side ventral horn showing CX3CR1-GFP microglia (**b1**), CD68 (**b2**) and superimposition of both (**b3**). CD68 is contained within CX3CR1-GFP microglia (arrowheads). (**c**) Confocal images of the ventral horn ipsilateral to the nerve injury and containing FB motoneurons and CX3CR1-GFP microglia (**c1**), CD68 (**c2**) and superimposition of both (**c3**). CD68 is upregulated in microglia around axotomized motoneurons (arrowheads).

After peripheral nerve injuries spinal cord microglia becomes activated in the dorsal horn projection areas of injured sensory afferents and in the ventral horn around the location of axotomized motoneurons (Fig. 1a1). In the ventral horn, microglia proliferate, migrate and cluster around axotomized motoneurons. In addition, activated microglia undergo changes in morphology from ramified to macrophage-like and this is parallel by many changes in gene and protein expression. Here we focus on CD68 (cluster differentiation 68), a member of the lysosomal/endosomal-associated membrane glycoprotein (LAMP) associated with macrophages and involved in phagocytosis and clearance of dead cells and extracellular materials. By difference to the lack of CD68 expression in resting/surveying rat spinal cord microglia probed with the rat-specific CD68 monoclonal antibody ED1, mouse microglia in the non-activated state show some basal expression of CD68, as revealed with the FA-11 monoclonal antibody. CD68 FA-11 immunostaining patterns were similar in the non-injured control side of the spinal cord after unilateral nerve injuries (Fig. 1a2) and in spinal cords of naïve uninjured animals. At high magnification, CD68 occurs in small round inclusions within “resting/surveying” microglia cell bodies and proximal processes (Fig. 1b). After activation, CD68 expression upregulates from basal expression levels (Fig. 1c).

To study possible microglia activation and development of phagocytic behaviors in “*ex vivo*” slices we first tested whether the slicing procedure by itself induced a phagocytic phenotype, or other phenotypes in microglia that could obscure the *in situ* responses of spinal microglia to nerve injury. We therefore analyzed microglia morphology and CD68 expression in ventral microglia at different times after obtaining slices from the spinal cord of a naïve CX3CR1-GFP mouse.

**Figure 2:**
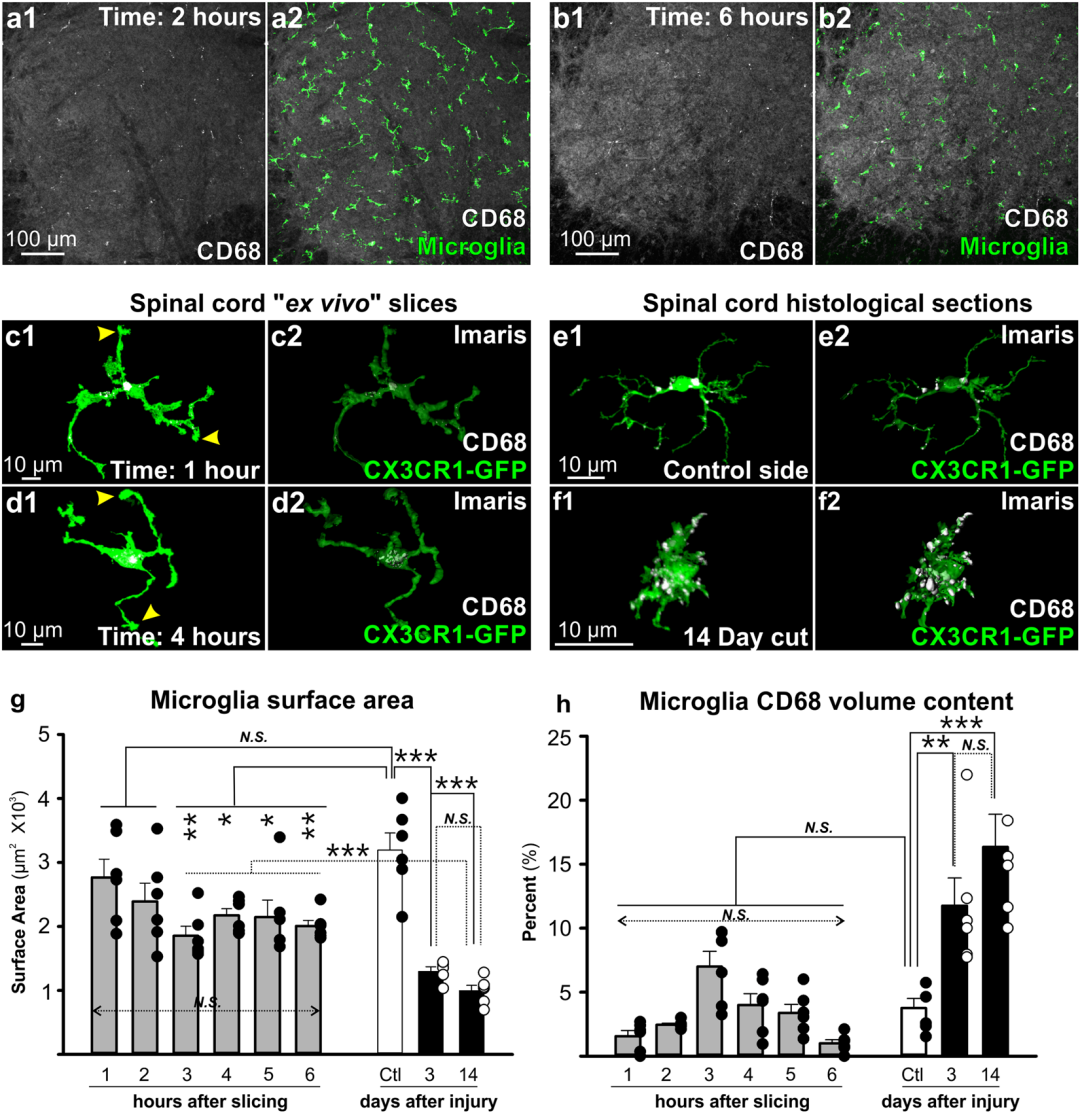
Validation of the slice preparation. (**a,b**) Low magnification confocal images of 50 µm thick optical stacks sampled >100 µm away from the section cut surface in 350 μm-thick spinal cord slices fixed at 2 (**a**) and 6 hours (**b**) after slicing (50 µm thick optical stacks sampled >100 µm away from the section cut surface). CD68 is in white and CX3CR1-GFP in green. (**c,d**) High magnification confocal images of single microglia (green) and CD68 content (white) 1 hour (**c**) and 4 hours after slicing (**d**). Each cell is shown as a 2D projection of the optical image stack (**c1,d1**) and in the Imaris 3D volume reconstruction (**c2,d2**). Processes of microglia in slices sometimes display club-like endings (yellow arrowheads). (**e,f**) High magnification confocal images of microglia from perfusion-fixed spinal cord sections in the control (contralateral, **e1,2**) and injured sides (ipsilateral, **f1,2**) 14 days after sciatic transection. Ipsilateral to the injury microglia have shorter processes, increased cell volume and higher CD68 content. (**g**) Average microglia surface area at different times after slicing (grey bars, n = 6 cells per time point, error bars = SE). No significant differences (N.S.) were detected among microglia collected at different times after slicing, but when compared with control (Ctl) perfusion-fixed microglia (white bar, n = 6 cells) significant differences were detected with microglia sampled from slices cut 3 to 6 hours before fixation (*p < 0.05, **p < 0.01). Microglia ipsilateral to the injury (black bars) showed large decreases in total surface area at 3 and 14 days after the injury (n = 6 cells per time point) and these were significant when compared to control and also to microglia 1 to 6 hours after slicing (***p < 0.001). (**h**) Percent of cell volume occupied by CD68 immunoreactivity within single microglia. There were no differences in CD68 content in uninjured animals at any time point after slicing, and CD68 content was similar to microglia from the control side of perfusion-fixed spinal cord sections. CD68 content significantly increased in microglia 3 and 14 days after injury (*p < 0.05, ***p < 0.001).

After the slices were cut (see Methods) they were immersion fixed (4% paraformaldehyde) one hour after sectioning or every hour thereafter up to six hours after slicing. Slices were analyzed with confocal microscopy after immunolabeling for CD68 and GFP (Fig. 2). Low-magnification confocal images demonstrated that ventral horn microglia in the “*ex vivo*” slices maintained their spacing without signs of clustering or proliferation up to 6 hours after slicing (Fig. 2a,b). Microglia in slices were fully reconstructed in Imaris from high magnification confocal images and their total surface area estimated (n = 6 microglia at each hour after slicing up to 6 hours, all from the same naïve spinal cord) (Fig. 2c,d). These cells were compared to microglia in the ventral horns of histological spinal cord sections located in control sides (n = 6 cells from 2 animals) or ipsilateral to a sciatic nerve injury, 3 and 14 days after lesion (n = 6 cells at each time point and each from a single animal) (Fig. 2e,f). Histological sections were obtained from perfusion-fixed animals and activated microglia were sampled from within the area of the lateral gastrocnemius (LG) motor pool, which was retrogradely labeled with Fast Blue ipsilateral to the injury (axotomized). Cell surface analyses revealed a decrease of 32% to 42% in total surface area in microglia 3, 4, 5, and 6 hours after slicing compared to control microglia in histological sections (one-way ANOVA, F(8, 53) = 9.76, p < 0.001; post-hoc Bonferroni t-tests comparisons to control: p = 0.002 vs 3 hours (t = 7.45), p = 0.043 vs 4 hours (t = 3.46), p = 0.033 vs 5 hours (t = 3.55)). There were no significant differences in surface area between microglia in slices fixed 1 and 2 hours after sectioning and control microglia in histological sections (p = 0.33 control vs 2 hours (t = 2.73); p = 1.000 control vs 1 hours (t = 1.45)). The decrease in microglia size in slices 3 or more hours after sectioning was significantly less than the changes in cell size recorded in microglia activated after nerve injury; 59% decrease 3 days after injury and 69% after 14 days (decreases estimated with respect to control microglia in the contralateral side). The surface areas of microglia in slices were always significantly larger compared to microglia activated 3 days after injury (p = 0.007 at 3 hrs; p = 0.009 at 5 hrs; p < 0.001 at 4 and 6 hrs, Bonferroni t-tests) and 14 days after nerve injury (in all cases p < 0.001 Bonferroni t-tests) (Fig. 2g). In summary, we found a partial reduction in cell size after slicing, but without developing typical macrophage-like morphology. These reductions in size might be due to partial retraction of distal processes. Microglia processes in slices frequently end as club-like endings (Fig. 2c,d) by difference to processes in resting/surveying microglia in histological sections (Fig. 2e). Cell bodies in slices were unchanged.

The lack of a phagocytic phenotype in the slice was confirmed by analyses of CD68 expression. CD68 content was estimated as a percentage of cell volume in the same microglia reconstructions. CD68 granules in control microglia (sampled from histological sections) occupied 3.75% ± 1.84 (S.D.) of total microglia volume. Average CD68 content of microglia sampled 1 to 6 hours after slicing ranged from 0.99% ± 0.70 to 7.00% ± 2.91 of total volume. In contrast, CD68 content inside microglia activated by nerve injury (sampled from histological sections) increased to 11.76% ± 5.30 at 3 days and 16.34% ± 6.25 at 14 days of the total cell volume (Fig. 2h). Differences were statistically significant between injured-activated microglia compared to controls or compared to microglia in slices (One-way ANOVA, F(8,53) = 16.365, p < 0.001; pair-wise Bonferroni post-hoc t-tests of microglia 14 days after injury compared to controls and 1, 2, 3, 4, 5, and 6 hours after slicing, all p < 0.001). No significant differences were found in microglia analyzed at different times after slicing or with control microglia in histological sections.

Thus, despite some small morphological alterations in distal processes we found no evidence of gross microglia activation or development of a phagocytic phenotype up to 6 hours after slicing (last time point analyzed). The data agrees with results in brain slices showing that microglia activation and expression of pro-inflammatory cytokines occurs only after 5–6 hours sectioning^51^. Similarly, microglia in hippocampal brain slices did not display morphological features of activation until 12 hours after slicing^52^. Importantly, in our two-photon time-lapse analyses, differences in microglia behaviors between activated (ipsilateral to the injury) and control states (contralateral to the injury) were not concealed or obscured by the slicing. Activated microglia displayed robust phagocytic behaviors and different dynamics compared to control microglia (see below). Control spinal microglia in the *ex vivo* slices displayed dynamics similar to cortical “surveying/resting” microglia visualized through cranial windows *in vivo*^42,53,54^. This also agrees with previous observations of control microglia in brain slices showing normal dynamic responses to exogenously applied ATP^51^. The findings align well with the fact that microglia can retain normal activity for long periods of time when faced *in situ* by anoxia and surrounding tissue death^43^.

**Figure 3:**
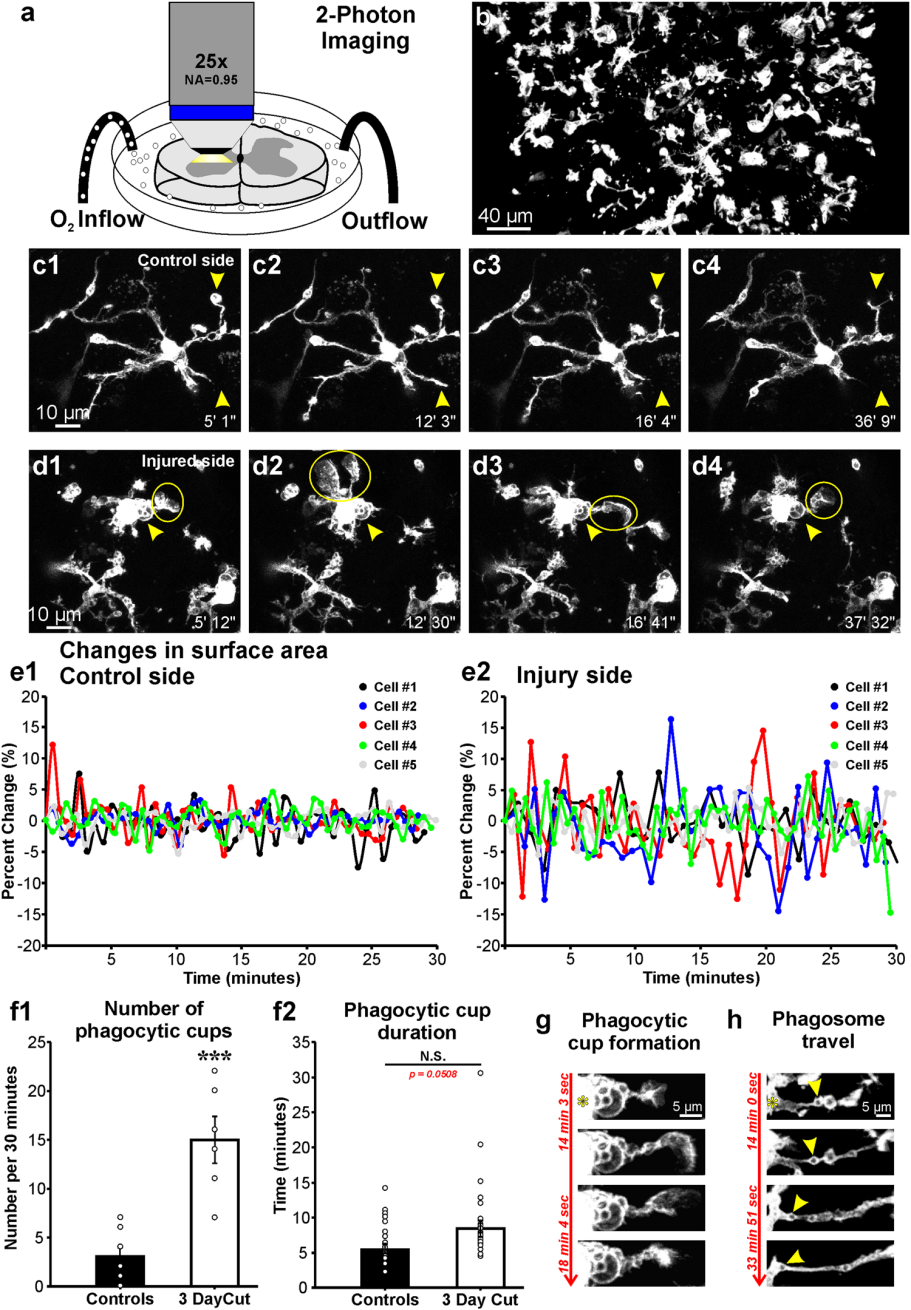
Microglia dynamics in adult spinal cord slices. (**a**) Slices were imaged with a 25X water immersion objective (0.95 NA). To avoid movement artifacts, bath circulation was stopped during imaging. (**b**) Single frame of Supplementary movie 1 showing the whole field of view of a ventral horn containing activated CX3CR1-GFP microglia (white) 3 days after a sciatic nerve cut. (**c1–4, d1–4**) Individual frames from 45 min time-lapse sequences of microglia contralateral (**c1–4**) and ipsilateral (**d1–4**) to the nerve injury (zoom = 3.5×). (c1–4 = 1 min 2.6 s between individual frames for a volume depth of 26 µm, d1–4 = 1 min 0.3 s between individual frames for a volume depth of 25 µm). Yellow arrowheads show positions of individual processes over time. Microglia ipsilateral to the injury are more active, expanding and contracting processes and forming phagocytic cups (yellow circles). (**c1–4)** frames are from Supplemental movie 2 and d1–4 frames from Supplemental movie 3. (**e1–2**) Changes in surface area (SA) over time (between each frame) in five control (**e1**) and five activated (**e2**) microglia. (**f1)** Average number of phagocytic cups per cell (n = 8 cells in control, black bars and n = 6 cells 3 days after axotomy, white bars) showed significant differences between control and 3 days after nerve injury (***p < 0.001). (**f2**) Average duration of individual cups formed by control and activated microglia (control: n = 21 cups, cut: n = 25 cups). Cup durations were similar except for a few parent processes in which full retraction were very slow. The difference did not reach significance (p = 0.0508). Error bars = SE. **g**) Formation and retraction of a phagocytic cup over approximately 4 minutes. Asterisk localizes the microglia cell body and it is surrounded by phagosomes. **h)** Example of a phagosome moving along a microglia process towards the cell body (yellow arrowhead). This frame sequence corresponds to Supplementary movie 4.

The results suggest that *ex vivo* spinal cord slices are adequate for studying microglia close to their *“in situ”* behaviors. To avoid possible late activation of microglia, all imaging was conservatively done within the first 4 hours after tissue slicing. Imaging through the cut surface of the slice (Fig. 3a) avoided image distortions caused by myelinated axons and improves resolution and sensitivity to optimally study microglia dynamics (Fig. 3b; supplementary movie 1). The slices represent a significant advance in image quality compared to previous *in vivo* imaging of spinal cord white matter microglia and allows for the first time visualization of microglia in the grey matter and their interactions with neuronal cell bodies.

### Microglia dynamics greatly differ ipsilateral and contralateral to the injury

Using time-lapse two photon microscopy we first imaged CX3CR1-GFP microglia in spinal cord slices from animals that had undergone a unilateral sciatic transection 3 days earlier. Ventral horn GFP microglia were imaged contralateral (control) and ipsilateral (activated) to the nerve injury. Individual microglia selected for analyses where located close to LG retrogradely labeled motoneurons. In these experiments we used Fast Blue, a fluorochrome that cannot be combined with GFP for dual color single excitation two photon microscopy, thus regions containing Fast Blue-labeled motoneurons were visualized with epifluorescence. We were careful to image microglia more than 50 µm away from the cut surfaces of the 350 µm-thick slices to avoid any interactions of the analyzed microglia with the damaged cut surfaces. Microglia in the contralateral side showed morphology and dynamics of surveying microglia extending and retracting individual processes at normal rates (Fig. 3c1–c4, supplementary movie 2). The one feature that differs from typical surveying microglia imaged *in vivo* was the presence of club-like endings in some processes. These club endings were dynamic, forming and dissolving with time. Their origin and significance are unknown, but they were also visible in microglia after fixing the slices and visualizing with routine confocal microscopy (see above and Fig. 2c1,d1). Activated ventral microglia located on the side ipsilateral to the nerve injury had shorter processes, larger cell bodies, and continuously extended phagocytic cups (Fig. 3d1–d4, supplementary movie 3). To quantify overall cell dynamics, individual microglia were surface-rendered using Imaris to compare the rate of total cell surface area change between microglia in spinal cord sides ipsi- and contralateral to the nerve injury. Control microglia retracted and elongated processes at matched rates resulting in little change in overall surface area. Process elongation and retraction were not as well matched in activated microglia, resulting in larger variations in total surface. Over a 30 minute imaging period, the change in surface area of control microglia ranged ±5% of the total surface, while in the activated state microglia surface change varied ±15%. The larger transient variations in total surface area in activated microglia are the compound result of the rapid extension and retraction of processes ending in phagocytic cups (peaks in surface variation usually coincide with the appearance of a phagocytic cup) and the fact that basal surface area is smaller in activated microglia. When the percent change was averaged over a 30 minute imaging period, control microglia change was 1.60% ± 0.24 (SE, n = 5 cells analyzed) while activated microglia change was 3.64% ± 0.53 (SE, n = 5). The difference was significant (t-test, t(8) = −3.493, p = 0.008) (Fig. 3e1–2), but the small effect sizes highlight the fact that over the 30 minute period there were no large drifts in the cell sizes of either activated or control surveying microglia. These results suggest that the “resting/surveying” and “activated” microglia morphologies we studied are relatively stable and that recording for 30–45 minutes, in our conditions, did not affect microglia function (longer imaging periods and stronger laser strengths, above 6%, caused the slow shrinkage of microglia and retraction of all processes, not shown).

A salient feature of microglia ipsilateral to the injury was the formation of phagocytic cups. Phagocytic cups extend outward from the cell body or from processes and produce a “claw-like” structure that appears to grab materials from the neuropil (Fig. 3d1–d4,g,h; supplementary movies 3 and 4). Microglia in the side contralateral to the injury (control) formed an average of 3.13 ± 0.89 (SE) cups per 30 minutes, while on the injured side microglia formed an average of 15.0 ± 2.30 (SE) cups during the same period (Fig. 3f1). The difference was significant (t-test, two-tailed t(12) = −5.374, p < 0.001; n = 8 microglia in control and 6 microglia on the injured side). Phagocytic cup duration was defined as the period from the initial appearance of a protrusion from the parent process until complete retraction of the cup back to the process and was sometimes longer for microglia ipsilateral to the injury, lasting on average 8.5 ± 1.6 (SE) mins in activated microglia compared to 5.6 ± 0.7 in the control side (Fig. 3f2). The difference, however, did not reach statistical significance (p = 0.0508; t-test, two-tailed t(44) = −2.008; n = 21 phagocytic cups recorded in 6 control microglia and 25 phagocytic cups recorded from 5 microglia in the injured side). The longer duration trend of phagocytic cups in microglia ipsilateral to the injury was related to their apparent larger size, however this was difficult to quantify given the fast dynamics of this structure compared to our sampling intervals during time-lapse recording. There was also significant diversity in the frequency and size of phagocytic cups in different microglia ipsilateral to the injury, even within the discrete regions of neuropil defined by single fields of view (Supplementary movie 3). Phagocytic cups in control microglia were less frequent and usually could be traced to processes extending towards the cut surface of the slice. For this reason, most of our analyses were done at depths of 50 to 150 µm from the cut surface. Nevertheless, spinal cord mouse microglia also express “*in situ*” a low steady-state level of CD68 (Fig. 1), suggesting low level phagocytic activity in the “resting/surveying” state, even without slicing.

The more intense phagocytic activity of microglia in the ventral horn ipsilateral to the injury parallels the upregulation of CD68 and the accumulation of phagosomes of larger size in microglia cell bodies and processes. Phagosomes move in a retrograde direction from distal processes towards the cell body (Fig. 3h, supplementary movie 4), where they accumulate. In conclusion, there are large differences in microglia dynamics between ventral horn microglia contralateral and ipsilateral to a nerve injury, with the latter expressing a phagocytic phenotype as early as three days after the injury.

### Microglia process dynamics differ between surveying and activated microglia, but terminal branch motility remains the same

**Figure 4:**
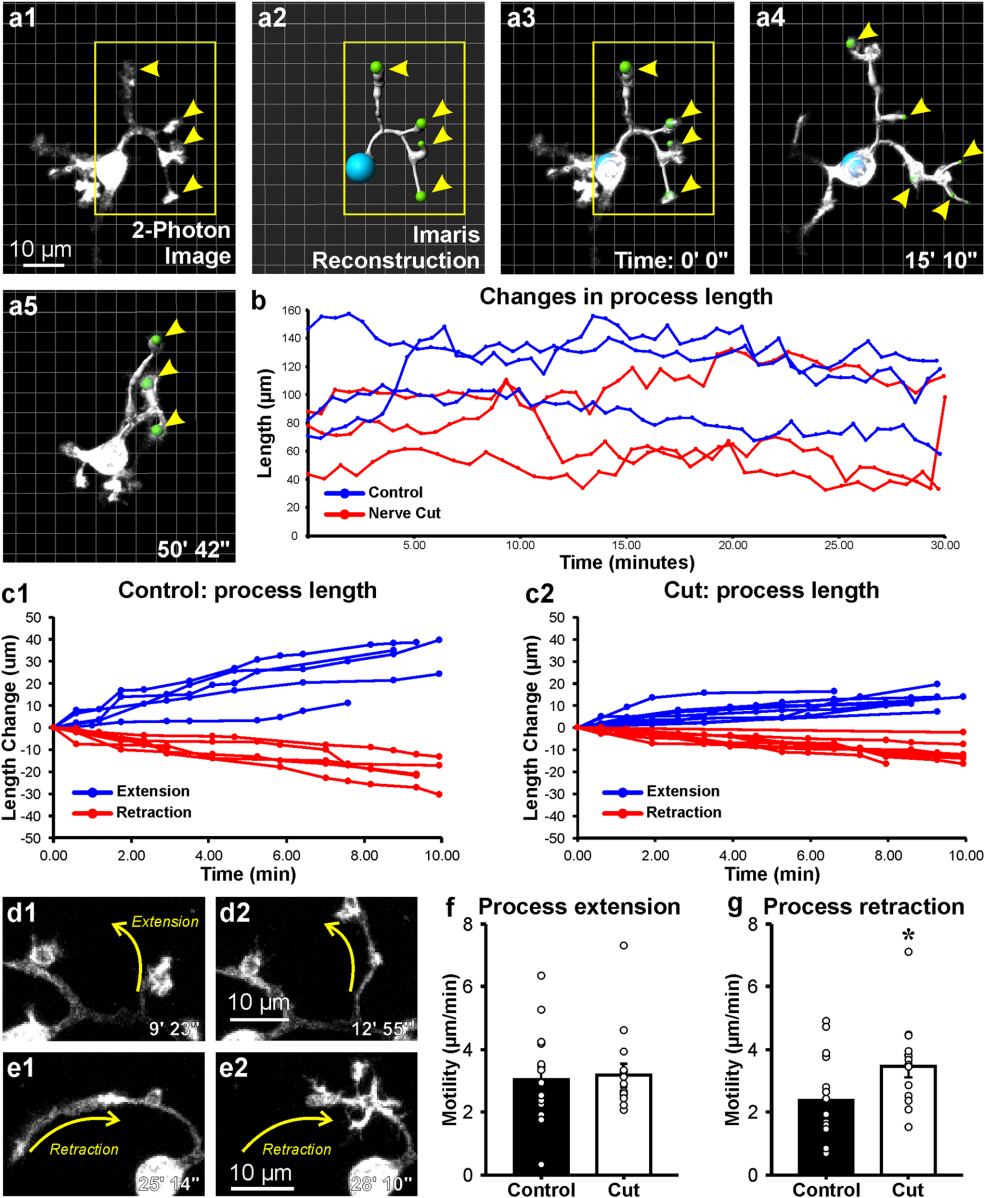
Individual microglia process motility. (**a1–5**) Control microglia reconstructed using Imaris filament tracker. Single process reconstruction in (**a2**). (**a3–a5**) superimposition of process reconstruction and two-photon images over time. All process filaments can be followed in supplemental movie 5. Yellow arrowheads identify examples of terminal branches used to calculate terminal branch motilities. (**b**) Overall change in process total length over time in control microglia (blue lines) and microglia responding to a sciatic nerve cut 3 days after injury (red lines). Each line represents one cell and the total length of its processes over time. Total process lengths are larger in control microglia compared to microglia responding to nerve injury. Activated microglia have shorter processes. (**c1–2**) Net change in extension (blue lines) and retraction (red lines) of individual processes from single control (**c1**) and activated microglia (**c2**). The changes in length represent total lengths and include the length of all branches in each process emanating from the cell body. The net change is greater in control for both extensions and retractions. (**d1–2, e1–2**) An example of terminal branch extension (**d1–2**) and retraction (**e1–2**). (**f,g**) Motility of extensions and retractions for terminal microglia processes (net change in length over time) in both control (black bars) and axotomized animals (white bars). Error bars are SE and each individual dot represents a single event (n = 14 events for both control and cut). Terminal process retraction was significantly slower in activated microglia (*p < 0.05).

Microglia process motility was quantified in the same cells analyzed above, but now reconstructed in 4D using Imaris filament tracker (Fig. 4a1–5, supplementary movie 5). We measured overall changes in total length for all processes (including all branches) stemming from the cell body of individual microglia (Fig. 4b: six microglia are shown; blue: naïve microglia; red: activated microglia ipsilateral to the injury, each line represents one cell and the total length of all its processes over time). We also estimated the change in length for individual processes and its branches (Fig. 4c1,2,c1, data from 5 processes from a control microglia; c2, data from 6 processes from an activated microglia). Finally, we estimated terminal-end motility calculated as a change in length of terminal processes from the last branch point to the end of the process (arrowheads show examples in 4a). Control microglia display overall longer total process lengths compared to microglia around axotomized motoneurons (Fig. 4b), as expected given their larger size. Within single microglia, individual processes display consistent changes in length over time when extending or retracting (Fig. 4c). Again, length extensions and retractions of individual processes were shorter in activated microglia. In this analysis, individual process lengths were estimated for each frame during a 10 minute time period and the change with respect to the previous frame calculated (each frame corresponds approximately to a 1 minute interval, see Fig. 4 legend). Length was measured from cell body to all endings, including all branches (Fig. 4a1–5). Thus, an increase in process length captures both branch elongation and the formation of new branches, while a decrease in length could be because the process shortens and/or withdraws branches. Processes changed from extending to retracting and vice versa during the 45–60 minute imaging period. Keeping analyses to a 10 minute time window allowed representation of continuous extensions and retractions in most processes, avoiding changes in directions or stops (exceptions are lines with no data before the 10 minute end-point in Fig. 4c1,c2; in this case the process stopped or changed direction earlier). The rates of change (slopes) during these 10 minute time windows were relatively constant for each individual process and higher for the larger processes of control microglia compared to the smaller processes of activated microglia ipsilateral to the nerve injury.

Terminal branch extension motility was similar in control and activated microglia (control: 3.04 μm/min ± 0.41 (SE); activated: 3.18 μm/min ± 0.37; two-tailed t-test, t(26) = −0.256, p = 0.7999, n = 14 per condition) (Fig. 4d1–2,4f, Supplementary Movie 2). However, there was a significant, increase in terminal branch retraction motility in microglia ipsilateral to the nerve cut (control: 2.38 μm/min ± 0.38, activated: 3.47 μm/min ± 0.36; two-tailed t-test, t(26) = −2.069, p = 0.048 (Fig. 4e1–2,4g). This change represents a 45.8% increase in the rate of retraction and it might be due to faster retractions of processes extending phagocytic cups. Overall, these velocities are all within the range of those described *in vivo* using cranial windows, although some differences occur depending on whether the analyses are biased towards terminal branch motility (faster) or overall process length change (slower)^42,53,54^.

Taken together with data on differences in surface change, the results demonstrate distinct microglia dynamics in surveying state around intact motoneurons compared to activated states in proximity to axotomized motoneurons.

### Interactions between microglia and motoneurons change with time after axotomy

**Figure 5:**
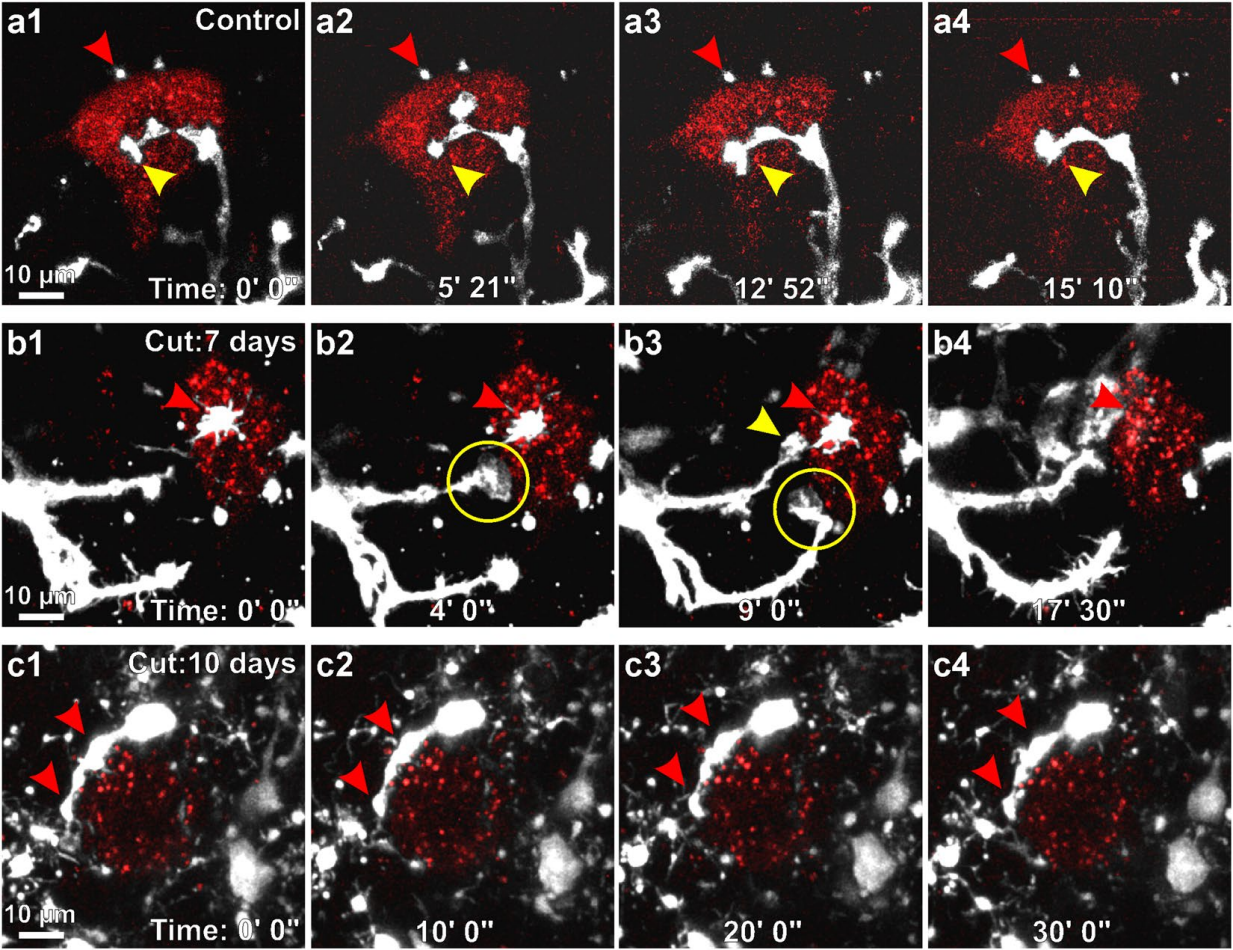
Microglia interactions with control and axotomized motoneurons. (**a1–4**) CX3CR1-GFP microglia (white) scanning the surface of LG motoneurons retrogradely labeled with CTB-555 (red) in a sham control animal (corresponds to Supplementary movie 6). Microglia send dynamic processes (yellow arrowhead) towards the motoneuron cell surface. Additionally, there are a few small microglia processes that make direct contact with the motoneuron and are stationary (red arrowhead). (**b1–4**) Microglia interactions with axotomized motoneurons 7 days post-injury (corresponds to Supplementary movie 7). At this time point, microglia extend their processes specifically towards the surface of the motoneuron (yellow arrowhead, (**b3**) at which point they form phagocytic cups close to the motoneuron surface (yellow circles). When the phagocytic cup disappears, the process is retracted. In addition, there are stationary microglia processes (red arrowhead) extending filopodia constantly scanning the motoneuron surface. (**c1–4**) Microglia covering the motoneuron surface 10 days after axotomy (corresponds with Supplementary movie 9). Microglia in this position are stationary and their cell bodies and processes (red arrowheads) are attached to the motoneuron surface. Filopodia lining microglia processes are active and in constant motion.

To visualize interactions between microglia and control or axotomized motoneurons, we injected the left LG muscle of CX3CR1-GFP heterozygous mice with the subunit b of Cholera Toxin coupled to Alexa 555 (CTb-555). This fluorochrome combination allowed for dual color two-photon microscopy using a single excitation wavelength (920 nm). One week after the CTb-555 injection, the sciatic nerve was either exposed and closed (sham) or exposed and transected (cut), and the spinal cord slice preparation was performed 7 or 10 days after surgery. We could not accurately image microglia-motoneuron interactions past 10 days post-injury as CTb-555 begins to be degraded by the motoneurons, making it difficult to distinguish cell body boundaries. In sham control mice, microglia processes do not surround motoneurons and make very few contacts with the cell body. The few processes in proximity of the cell body extend filopodia at the motoneuron surface and then retract (Fig. 5a1–4, supplemental movie 6). Following nerve injury, microglia increase their rate of interactions with motoneuron cell bodies, and the type of interactions evolve from 7 to 10 days after injury. Seven days after injury, most microglia cell bodies were located at a distance from retrogradely labeled LG axotomized motoneurons and continuously extend and retract processes towards the motoneurons, making contacts that frequently end in small brief phagocytic cups (green circles in Fig. 5b1–4, supplemental movie 7). Some microglia processes appeared attached to the motoneuron cell body for long periods of time and continuously extended filopodia over the motoneuron surface (red arrow Fig. 5b1–4 and supplemental movie 7). Ten days after injury, the cell bodies of axotomized motoneurons were surrounded by microglia that have attached their cell bodies to the motoneuron surface. Microglia in this position moved little and did not extend or retract processes, however they constantly scan the motoneuron surface with filopodia that emerge from stationary processes (Fig. 5c1–4 and supplemental movie 8).

**Figure 6:**
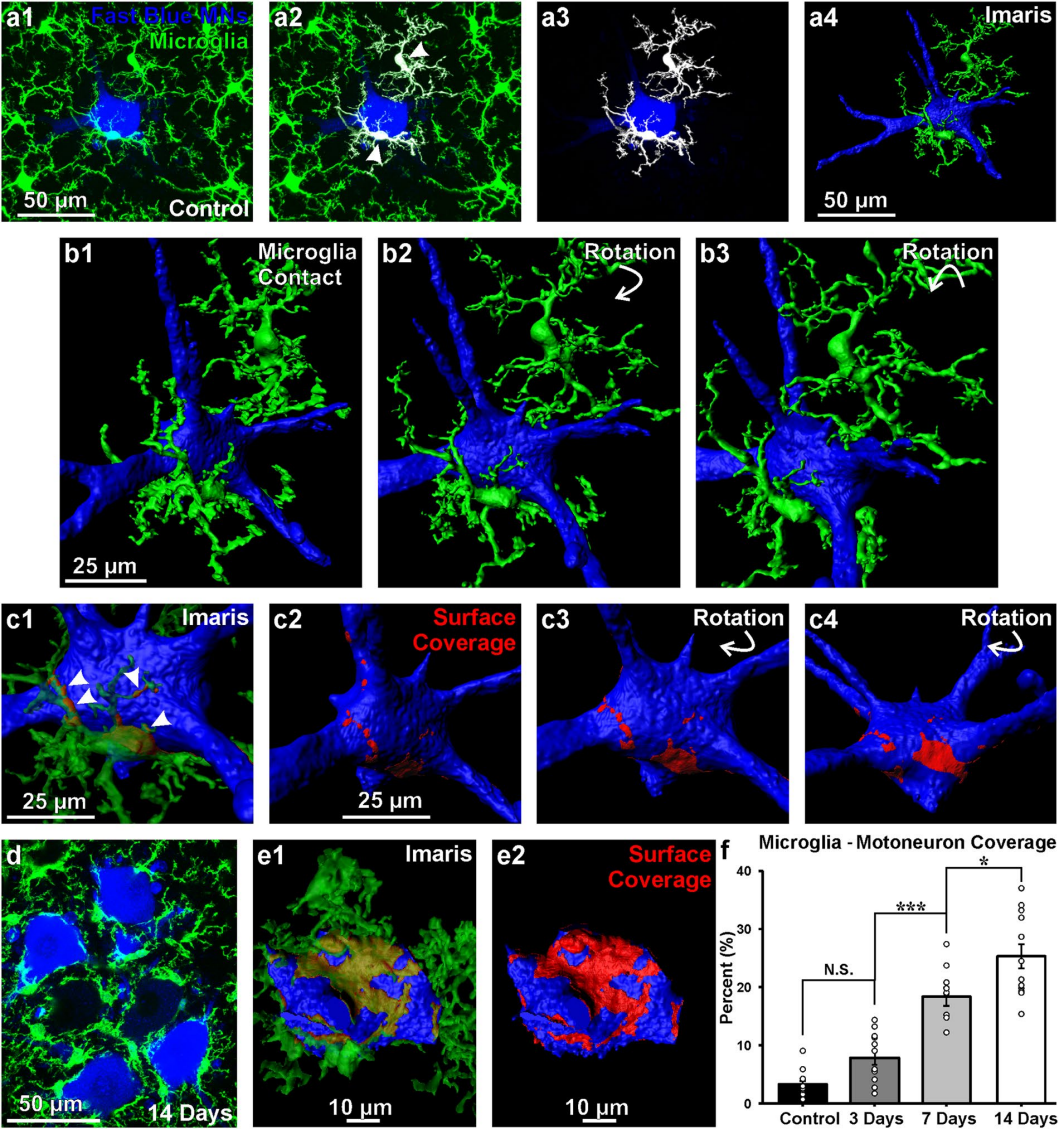
Motoneuron surface is increasingly covered by microglia with time after axotomy. (**a1–4**) Confocal images and Imaris image segmentation of connected cells. (**a1)** 2D projection of a Fast-Blue retrogradely labeled motoneuron and surrounding CX3CR1-GFP microglia in a sham control animal. (**a2,3)** Imaris image segmentation of microglia in contact with the Fast Blue labeled motoneuron cell body (white cells in a2) followed by their isolation (a3). (**a4)** Surface rendering of the motoneuron and associated microglia. (**b1–3**) High magnification of reconstructed cells shown at various rotations. (**c1–4**) Isolation of points of contact. The area detected with distance “0” between motoneuron and microglia surfaces were identified (arrowheads) and rendered (red in **c2**). The motoneuron surface covered by microglia processes is shown in two further rotations (red in **c3,c4**). (**d**) 2D projection of Fast-Blue labeled axotomized motoneurons covered by activated microglia 14 days after the injury. (**e1**) Surface rendering of one motoneuron cell body and all the attached microglia. (**e2**) Motoneuron surface covered by microglia processes (red). (**f**) Quantification of the percentage of motoneuron surface covered by microglia. Each dot represents an individual reconstructed motoneuron (n = 10–12 MNs per condition). N.S. non statistical significant trend towards increase surface coverage from sham controls to 3 days post-injury. ***p < 0.001 and **p < 0.01 statistical significant increases in surface coverage from 3 to 7 days after injury and 7 to 14 days after injury. Supplementary movies 9 and 10 show respectively microglia surface coverage of control and axotomized motoneurons.

CTb-555 retrograde labeling appeared as intracytoplasmic granules that did not uniformly fill the cell body, preventing us from obtaining accurate estimates of surface coverage. To better quantify the evolution of motoneuron-microglia surface coverage with time after injury, we retrogradely labeled LG motoneurons with Fast Blue and obtained histological sections from perfusion-fixed spinal cords (Fig. 6a1). Fast Blue completely fills the cell body and is more stable inside the motoneuron with no loss in fluorescence with time after injury, however it is not visible while performing single wavelength two-photon excitation in the CX3CR1-GFP mice. Using confocal microscopy we improved resolution of the motoneuron surface at different times post-injury and accurately defined contact regions with microglia (Fig. 6a2–4). The sections were imaged at high magnification (60X NA 1.35) and analyzed by 3D rendering their surfaces in Imaris (Fig. 6, supplemental movies 9 and 10). These analyses were performed in animals that carried both CX3CR1-GFP and CCR2-RFP alleles (see methods). The CCR2-RFP allele allowed us to define immune cells that infiltrate the spinal cord from the blood, as described previously^30^.

We found a significant increase in coverage of the motoneuron cell body surface by CX3CR1-GFP cells from 3 to 14 days after the injury. We quantitatively analyzed 10–12 randomly selected motoneurons in sham controls and 3, 7 and 14 days after injury. Motoneuron-microglia contacts were defined as regions with nominal “0” distance between both surfaces in three-dimensional space (see methods) (Fig. 6c1–4). The percentage of motoneuron surface covered by microglia increased from sham controls to 3 days after injury, although the difference was not significant. There were significant increases in motoneuron surface covered from 3 to 7 days and from 7 to 14 days after axotomy (Fig. 6d,e1–2,f) (one-way ANOVA, F(3, 42) = 47.53, p < 0.001; Bonferroni post-hoc, control – 3 days, p = 0.177, 3 days – 7 days, p = <0.001, 7 days – 14 days, p = 0.013).

**Figure 7:**
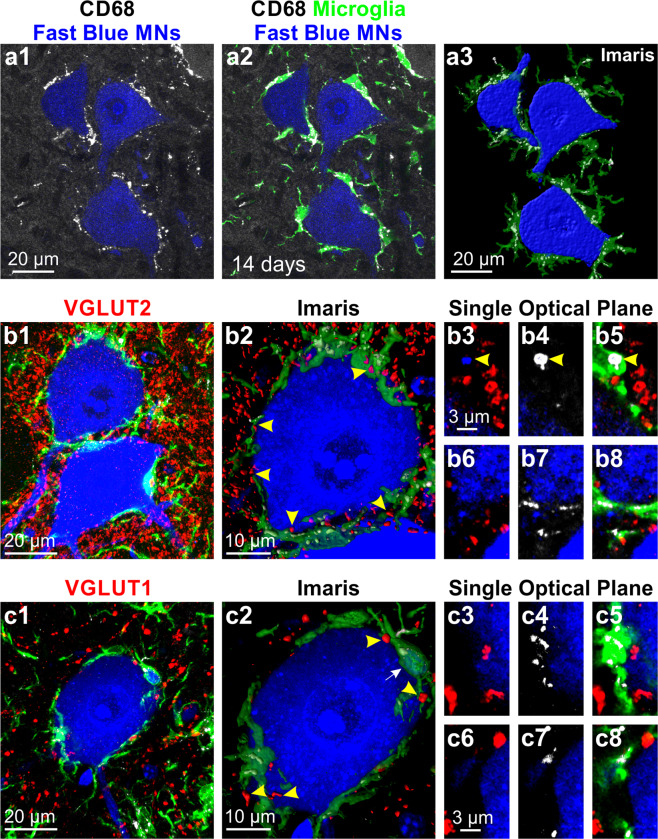
CD68 granules concentrate at the microglia surface interacting with axotomized motoneurons but do not engulf excitatory synapses. (**a**) High magnification confocal image of LG Fast Blue labeled motoneurons 14 days after axotomy showing CD68 immunoreactivity surrounding the cell bodies (white) in (**a1)**. This CD68 –IR granules are inside microglia processes opposite to the motoneuron surface (**a2**, green CX3CR1-GFP). (**a3**) Volume rendering of the motoneurons, apposed microglia and CD68 granules contained within the microglia processes. (**b1,c1)** High magnification confocal images of Fast Blue labeled motoneurons 14 days post injury surrounded by Iba1+ microglia (green) with high density of CD68-IR granules (white) in combination with excitatory synaptic markers VGLUT2 (**b1**) and VLGUT1 (**c1**). Each collapsed confocal image stack contains 15 image planes taken at 0.5 µm z steps. (**b2, c2)** Zoomed Imaris volume renderings of motoneuron cell bodies and microglia with close by VGLUT2 (**b2**) and VGLUT1 ( **c2**) synaptic boutons. Yellow arrows show VGLUT synaptic inputs close to microglia, but always outside. White arrow in c2 indicates a microglia cell nucleus filled with Fast Blue content. There was no clear evidence of reactive microglia engulfing excitatory inputs or of CD68+ granules incorporating VGLUT synaptic material. (**b3–b8, c3–c8)** Single optical planes of the confocal images stacks from b1 and c1 show no co-localization between VGLUT2 or VGLUT1 and CD68 granules. Yellow arrowhead in b3–b5 show a CD68+ phagosome containing Fast Blue.

**Figure 8:**
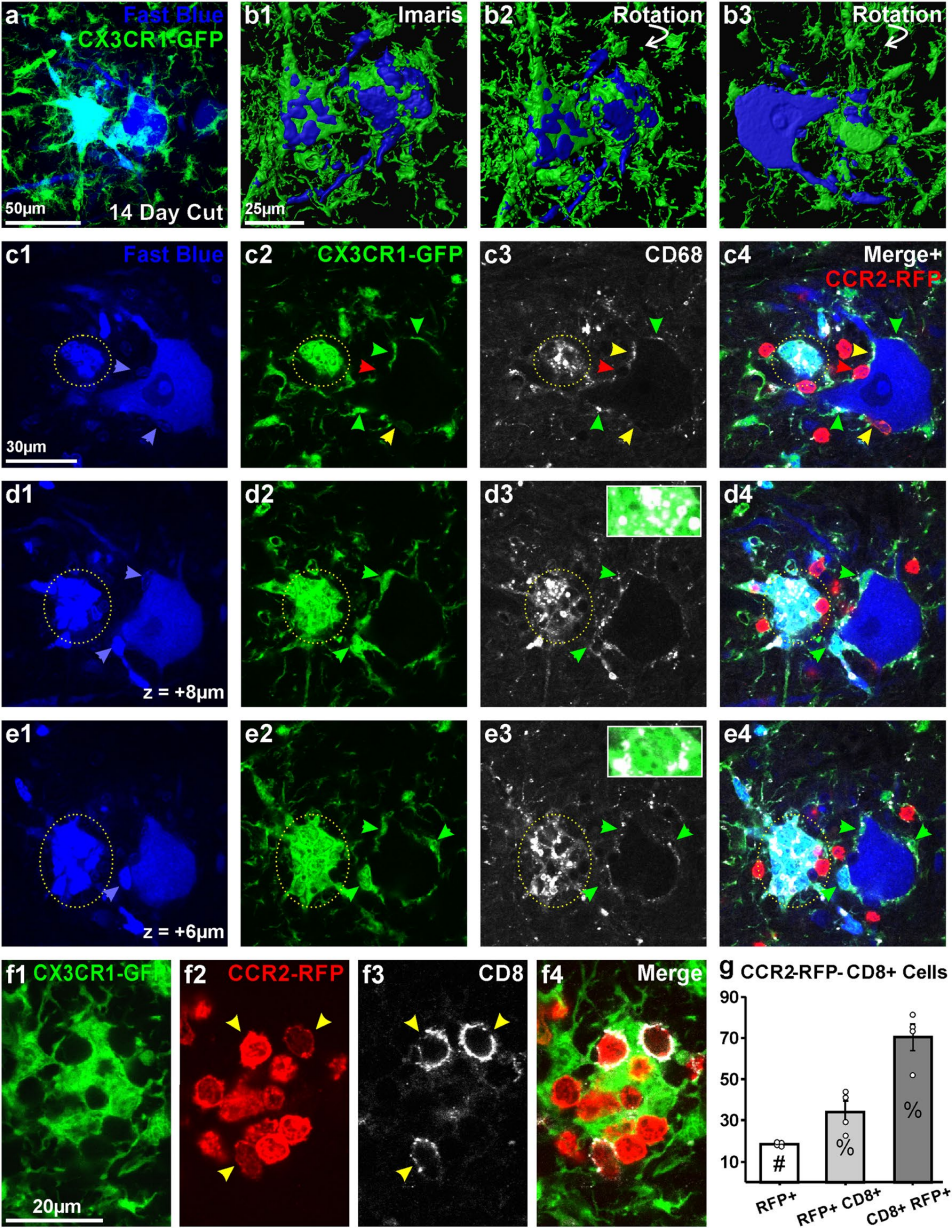
Macroclusters of microglia and CD8 cytotoxic T-lymphocytes surrounding degenerating motoneurons. (**a**) Confocal image through two Fast Blue motoneurons with different microglia coverage 14 days after axotomy. The left motoneuron is fully covered by CX3CR1-GFP microglia; the one in the right is only partially covered. (**b**) Same cells surface rendered in Imaris. (**b1)** top surface of both cells; (**b2)** partial rotations; (**b3)** 180° rotation showing mid cross-sectional planes of these cells. The motoneuron in the left (right in b3 after 180° rotation) has a blebbed cytoplasm and is degenerating. The motoneuron in the right (left after 180° rotation) shows normal cell body and dendrites. (**c1–4;d1–4;e1–4**) Single confocal optical planes of both motoneurons (**c1,d1,e1**), CX3CR1-GFP microglia (**c2,d2,e2**), CD68 (**c3,d3,e3**) and three colors superimposed with addition of CCR2-RFP cells (**c4,d4,e4**). Z values in d1 and e1 are respectively the increased section depth from c to d and then from d to e. The dotted circle indicates the microglia macrocluster. Microglia are tightly anastomosed to each other within the macrocluster and fully cover the degenerating motoneuron. Microglia inside the macrocluster highly express CD68 (**e3,d3,e3**). Microglia attached to the “healthy” motoneuron incorporate Fast Blue (blue arrows in **c1**, **d1** and **e1)**. These correspond to CX3CR1-GFP microglia (green arrowheads, c2–4, d2–4, e2–4), CCR2-RFP cells (red arrowheads) or dual genetically labeled cells (yellow arrow). CD68 is expressed in all these cells surrounding the motoneuron. CD68 expression is higher in CX3CR1-GFP cells. (**f1–4**) CCR2-RFP cells in CX3CR1-GFP microglia macroclusters. Some express the cytotoxic T-cell marker CD8. (**g**) CD8 in CCR2-RFP cells. White bar indicates average number of CCR2-RFP cells in the ventral horn per 50 µm thick section. Gray bars are percentages of CCR2-RFP cells that are CD8 + and CD8 + cells that are CCR2-RFP. Each dot represents one animal (n = 4). Error bars represent SE.

Immunolabeling with CD68 revealed that microglia surfaces opposed to motoneuron cell bodies 14 days post-injury contain a high density of CD68 granules (Fig. 7a). Co-labeling with the excitatory synapse markers VGLUT1 and VGLUT2 in quadruple fluorescent images (Fast Blue motoneurons, Iba1-IR microglia, CD68-IR and either VGLUT) demonstrated that CD68 granules never incorporated synaptic material (Fig. 7b,c). This was confirmed in both volume reconstructions of microglia and CD68 granules (Fig. 7b2 and c2, and supplementary movies 11 and 12) and by analyzing CD68 and VGLUT co-localization in series of single optical confocal sections (Fig. 7b3–8 and c3–8 and supplementary movies 13 and 14). Similar results were obtained when using a marker for inhibitory synapses (VGAT, not shown). Some microglia incorporated Fast Blue, suggesting transfer of motoneuron contents to surrounding microglia (Figs. 7b,c and 8c1,d1,e1, blue arrowheads). Fast Blue was usually found in the nucleus of microglia (Fig. 7c, arrow) or more diffusely throughout the surface of apposition of microglia and motoneurons (Fig. 7b1, bottom motoneuron). In some instances large CD68 granules inside microglia accumulated Fast Blue (Fig. 7b3). However, most CD68 granules inside microglia did not localize with Fast Blue or synaptic markers and their exact content needs to be further investigated.

Based on CX3CR1-GFP and CCR2-RFP labeling three different types of cells were found around motoneuron cell bodies: CX3CR1+/RFP+, CX3CR1−/RFP+ and CX3CR1+/RFP− (respectively yellow, green and red arrowheads in Fig. 8c2–4,d2–4,e2–4). RFP+ cells were interpreted previously^30^ as infiltrated peripheral CCR2+ monocytes transforming into branched microglia-like cells while upregulating CX3CR1-GFP and downregulating CCR2-RFP. CD68 was more strongly expressed by cells with intense CX3CR1-GFP; less CD68 was detected inside CCR2-RFP+ cells with weak or no CX3CR1-GFP. All cell types were however capable of incorporating Fast Blue.

Most motoneurons surrounded by CX3CR1-GFP and CCR2-RFP cells did not show morphological features of degeneration. However, a few were tightly covered by macroclusters of anastomosed microglia completely covering the motoneuron cell body (Fig. 8a,b). In this case microglia massively upregulated CD68 and the motoneuron found underneath showed morphological signs of degeneration (Fig. 8c–e). Round CCR2-RFP cells lacking processes were also embedded within CX3CR1-GFP microglia macroclusters. Many of these CCR2-RFP cells expressed the cytotoxic T-cell marker CD8 (Fig. 8f). CD8+ cells represent 34.2± 5.1% (SE) of all CCR2-RFP cells present in the ventral horn area containing axotomized motoneurons, and 70.4 ± 6.4% of CD8+ cells were CCR2-RFP (Fig. 8g). In conclusion, microglia associated to degenerating motoneurons display a distinct phenotype: they form microglia macroclusters, express high levels of CD68 and co-localize with CD8+ T-cells.

## Discussion

This work describes for the first time the behavior of microglia around motoneurons axotomized by injuries to peripheral nerves. Microglia in the proximity of axotomized motoneurons are known to proliferate, change morphologies, upregulate a number of cytokines and neurotrophic factors, and migrate towards the cell bodies of motoneurons eventually surrounding them^1,4,19,55–59^. Despite the abundance of data on the properties and time course of the microglia reaction, the exact function of activated microglia around axotomized motoneurons is not fully understood. Microglia wrapping of motoneurons has been associated with synaptic stripping and neuroprotection^1,4,19,55–57,59^, but these conclusions are challenged in other reports^19,22,30,60–62^. The two-photon and confocal analyses we describe suggest that a variety of behaviors can be displayed by microglia around axotomized motoneurons and these might correlate with a multiplicity of roles, sometimes with opposing functions on adjacent motoneurons. Microglia associated with axotomized motoneurons express a phagocytic phenotype that is quite distinct from microglia in the resting surveillance state. However, phagocytic cup formation rates, phagosome and CD68 content, and morphology can all vary from cell to cell, and these behaviors change with time after injury. An extreme phenotype occurs when microglia associate with degenerating motoneurons. In this case CD68 content is maximal, microglia form macroclusters and also associate with cytotoxic T-cells. Further diversity derives from CX3CR1-GFP microglia originating from either resident microglia or infiltrating CCR2 monocytes. The following discusses some salient features found in the behaviors of CX3CR1-GFP microglia around motoneurons after axotomy.

### Phagocytic activity of microglia in the vicinity of axotomized motoneurons

Whether activated microglia around axotomized motoneurons are phagocytic was controversial. Phagocytic markers, like CD68 (recognized with the monoclonal antibody ED1 in rats), were not found in microglia around adult facial motoneurons axotomized after a distal nerve injury^58,63^. CD68 was however found upregulated after proximal axotomies and in neonatal animals, both manipulations inducing extensive motoneuron cell death. Moreover, microglia around axotomized motoneuron cell bodies examined with electron microscopy were never found engulfing synapses^1,7^. These results lead to the often repeated conclusion that the functional state of microglia after nerve injury does not include a phagocytic phenotype^64^. However, studies in rat spinal motoneurons comparing distal (sciatic nerve) and proximal (ventral root avulsion) motor axon injuries detected upregulation of ED1-immunoreactive CD68 in microglia around spinal motoneurons after both injuries, although CD68 expression almost doubled after ventral root avulsion (inducing extensive cell death) compared to sciatic nerve injuries (inducing limited cell death)^34,39,65^. Similarly, we found CD68 content higher in microglia associated with degenerating motoneurons, but it was also upregulated in microglia surrounding healthy motoneurons. Upregulation of CD68 is frequently interpreted as development of macrophage function and our two-photon time-lapse analyses confirmed this behavior. Microglia around axotomized motoneurons were highly phagocytic. At 3 and 7 days after injury, most microglia had cell bodies at a distance from motoneurons and extended phagocytic processes that seemed to remove materials close to the motoneuron surface. By 10 days after injury, microglia attached and wrapped the motoneuron cell body. In this position microglia did not extend phagocytic cups but express numerous CD68 granules concentrated to the cytoplasmic side opposite to the motoneuron surface.

The abundance of phagocytic cups visualized with two-photon microscopy during the first week after injury contrasts with the low frequency of recognizable phagocytic cups in activated spinal microglia analyzed in fixed tissues. The reason for this is unclear. Slicing did not induce strong phagocytic phenotypes in naïve microglia, however a recent study found that anoxia induced during the vascular rinse prior to perfusion-fixation alters the morphology of CX3CR1-GFP microglia branches^66^. Anoxia could also be detrimental to phagocytic cup formation. Given the transient nature of phagocytic cups, a decrease in their rate of formation during perfusion could explain their low frequency in fixed-tissue sections and highlights the need of two-photon real time imaging to fully understand microglia behavior.

An important question is the significance of this evolving phagocytic activity. Previous lack of electron microscopy evidence for microglia engulfment of synapses on the surface of axotomized motoneurons and the fact that microglia are not necessary for synaptic stripping discards somatic synapses as possible targets^1,19–21,30^. VGLUT1 synapses originating in muscle spindle sensory afferents (also injured in the nerve) may be an exception, as we found that microglia activation is required for their permanent removal^30^. But the removal of these synapses occurs throughout the ventral horn and it is not focused on microglia interactions with the cell body surface. Despite being synapses frequently located close to microglia, we could not find any incorporation of synaptic materials inside CD68 granules or clearly inside microglia. This is in agreement with extensive previous electron microscopy literature that was recently reviewed^67^ and that consistently showed that microglia does not phagocytize the synapses that detach from the cell bodies of axotomized motoneurons. On the other hand, the recently discovered microglia-dependent mechanism that permanently removes VGLUT1 synapses and axons throughout the spinal cord ventral horn after the peripheral branches of these sensory axons are axotomized following nerve injuries has not yet been fully clarified^30^.

Alternatively, microglia could be removing extracellular matrix, in particular perineuronal net (PNN) components that normally encase the cell bodies of adult motoneurons^68,69^. PNNs detected by the binding of *Wisteria floribunda* lectin to chondroitin sulfate proteoglycans partially dissolve around axotomized spinal motoneurons two weeks after sciatic nerve injury^70^. Tenascin-R is a major PNN component with anti-adhesive properties for activated microglia and tenascin-containing PNNs are partially removed by the combined action of degradation by microglia-secreted factors and expression downregulation in motoneurons after injuries to facial or hypoglossal nerves^62^. Interestingly, tenascin-R PNNs surrounding axotomized motoneurons are preserved in cathepsin-S knockout mice, an enzyme that degrades PNN proteoglycans and extracellular matrix. Tenascin-R preservation was associated with diminished microglia translocation to the motoneuron cell body surface^23^. It is thus possible that PNN degradation is a necessary first step for microglia gaining access to the motoneuron surface. Partial degradation of motoneuron PNNs has been proposed to increase synaptic plasticity^70^ and to be neuroprotective^23^; more motoneurons were degraded in cathepsin-S KO mice lacking microglia enwrapping of motoneurons. Thus, the early phagocytic activity of microglia on the surface of motoneurons might be interpreted as a mechanism to remove PNN material and facilitate microglia access and attachment to the cell bodies of axotomized motoneurons.

### Properties of microglia enwrapping healthy motoneurons

Two weeks after sciatic injury microglia enwrap axotomized motoneurons. In this position, microglia processes move little, do not extend phagocytic cups but continuously scan the motoneuron surface extending and retracting filopodia. CD68 granules in the microglia cytoplasmic side facing the motoneuron, suggest active microphagocytosis. Similar behavior was recently described over excitatory synapses on hippocampal neuron spines and was named trogocytosis^71^. During this process, small areas of the plasma membrane (<1 µm) are invaginated and pinched off by attached microglia processes expressing CD68. The authors indicated that this was exclusively observed on presynaptic bouton surfaces and never on postsynaptic structures. Our data suggest a similar process might be occurring on the postsynaptic motoneuron surface after axotomy. Incorporation in microglia of motoneuron intracytoplasmic content is also suggested by the transfer of Fast Blue. Some of this material was found inside CD68 granules, but most Fast Blue accumulated in the microglia nuclei.

Filopodia and trogocytosis might be two mechanisms by which attached microglia sample the extracellular and intracellular composition of axotomized motoneurons, perhaps monitoring motoneuron health. Early studies that reduced motoneuron cell body attachment by blocking microglia proliferation with mitotic suppressors found no increase in cell death in axotomized hypoglossal motoneurons^22^. However, other studies in the facial nucleus showed increased cell death in transgenic mouse models that reduced, through a variety of mechanisms, the extent and strength of microglia-motoneuron attachment (GFAP-IL6 mice^72^; cathepsin KOs^23^; TGFβ1 KOs^73^). In particular, neuroprotection of facial motoneurons was found to result from a fine interplay between microglia and CD4 T-cells regulating local astrocyte function^32^, being astrocytes ultimately responsible for exerting neuroprotection via upregulation of STAT3 and expression of trombospondin-1^35^. Microglia attachment to the motoneuron cell body surface might regulate signaling in these processes.

Microglia wrapping of cortical neurons after traumatic brain injuries was also found to correlate with enhanced neuroprotection and decreased expression of neuronal stress markers^74^. In this case, enhanced neuroprotection was explained by microglia displacement of inhibitory synapses and increased activity in injured neurons^18^. Whether these results inform microglia function on axotomized motoneurons is unclear. Synaptic stripping on spinal motoneurons after sciatic nerve injuries affects predominantly glutamatergic and less GABA/glycinergic synapses^24^. Moreover, retained inhibitory synapses likely become excitatory because downregulation of KCC2 shifting chloride flow in GABA_A_ and glycine receptors to an outward current^31^. GABA/glycine synapse remodeling and KCC2 downregulation on axotomized spinal motoneurons are both independent of microglia^30,31^. Therefore, any neuroprotective effect of microglia over surrounded motoneurons is likely to operate through mechanisms other than inhibitory synapse removal.

Neuroprotection might depend on microglia detection of a variety of “non-eat” signals through cell-to-cell contact^75^. In addition, activated microglia upregulate a large number of neurotrophic factors that can act directly on the motoneuron in a paracrine fashion. Close proximity between both cells would facilitate this function. Finally, microglia-derived cytokines modulate a neuroprotective astrocytic response. It is well known that with time after axotomy (>2 weeks) microglia are gradually substituted by astrocytes over the surface of axotomized motoneurons^67^.

### Microglia macroclusters and their role in motoneuron cell death

Microglia formed macroclusters that completely surround motoneurons when these were undergoing degeneration (cell body shrinkage, membrane blebbing, varicose dendrites, etc.). These microglia express maximal CD68, matching the levels reported in similar “nodules” or “clusters” after spinal cord injury and believed to remove cellular debris^76^. By difference to the abundance of microglia clusters throughout gray and white matter after spinal cord injury, microglia macroclusters after nerve injuries are circumscribed to just the cell bodies of a few dying motoneurons.

Whether microglia macroclusters represent phagocytosis of cellular debris from apoptotic cells or live motoneuron phagoptosis^75,77^ will require capturing the process with two-photon live microscopy. In the present experiments this was not possible due to two limitations. First, these events occur at low frequency. Thus, they were best detected by searching serial histological sections throughout the lumbar spinal cord and were never captured in the slices imaged with two-photon microscopy. The low probability of finding such macroclusters is the consequence of limited cell death (estimated to be around 10% of motoneurons in adult mice spinal cords after distal nerve injuries^78^) and the likely protracted period through which cells die. Microglia macroclusters over degenerated Fast Blue LG motoneurons were observed at 7, 14, and 21 days post-injury. Second, the time required for phagocytosis of full neurons is likely longer than our recording time-lapse period; while phagocytosis of abnormal retinal photoreceptors occurs within 20–30 minutes^79^, removal of full neurons by phagoptosis takes several hours in cell cuture^80^. Similarly, swarming of microglia around neurons targeted for deletion in Alzheimer disease models lasts several days^81^. Future experiments will require increasing the incidence of motoneuron cell death (by performing ventral root avulsion instead than distal nerve injuries, for example) and extending the imaging period to capture longer time segments of the process. Nevertheless, the involvement of cytotoxic T lymphocytes suggests that microglia macroclusters represent a death microenvironment for motoneurons. Similar T-cell and microglia nodules occur after virus infection, encephalitis and multiple sclerosis, and they were postulated to phagocytose cells being actively killed by cytotoxic T lymphocytes^82,83^. Which motoneurons are targeted for such removal and the nature of the signals leading to microglia clustering and T-cell recruitment are unknown. In any case, this represents a novel mechanism of motoneuron cell death after axotomy, in addition to cell-autonomous necrotic and apoptotic mechanisms described in neonates or after proximal nerve injuries in adult^38,39^.

### A new adult slice spinal cord preparation for *ex vivo* two-photon microscopy imaging of microglia dynamics

The new *ex vivo* imaging technique described here allows visualization of microglia in spinal cord regions not readily visible through spinal windows. With the appropriate genetic reporters and/or tracers, this method will permit investigators to explore the interactions of microglia in adult spinal cords during synaptic removal, neuronal protection or neuronal death in health and disease. The strength of this preparation is highlighted by comparison to a recent publication describing a method to visualize the ventral horn *in vivo* using two photon microscopy of CX3CR1-GFP microglia combined with retrogradely labeled motoneurons^49^. As indicated by the authors in the discussion of this paper, “*the surgery requires substantial training… It is the combined effort of getting access to the ventral spinal cord, minimizing surgery-dependent bleedings and maintaining vital body functions of the mouse. In particular, bleeding has to be prevented to avoid blurring image recording. Despite optimization efforts, due to the overlaying 200 µm of white matter light scattering will still impede the visualization of thin processes.”* Our preparation overcomes these problems, and we demonstrate that the behaviors observed are representative of those “*in situ*”. First, there was a clear difference between microglia ipsilateral and contralateral to the injury. Second, we found no upregulation of microglia phagocytic phenotypes in spinal cord slices obtained from control uninjured mice. Third, microglia dynamics were similar to those observed *in vivo* through cranial nerve windows^42,53,54^. A thorough review of the challenges for *in vivo* imaging deep in the spinal cord was recently published^84^.

In summary, we characterized an *in vitro* adult slice preparation to study interactions between microglia and neural elements in the deep ventral horn of the spinal cord with high spatial and temporal discrimination. Using this preparation, we found that microglia in the vicinity of axotomized motoneurons become phagocytic and display evolving behaviors regulated with time after injury. Early after axotomy they remove components from the motoneuron cell body surface, thereafter they attach to the cell body and screen the intracellular and extracellular composition of motoneurons perhaps monitoring health. Over degenerating motoneurons they swarm forming macroclusters associated with cytotoxic T-cells. The results suggest a number of different functions for activated microglia around axotomized motoneurons.

## Materials and Methods

### Animals

All animal experiments were approved and complied with Emory University’s Institutional Animal Care and Use Committee (IACUC). All methods were performed in accordance with the NIH guidelines and Public Health Services regulations for the Human Care and use of laboratory animals.

Adult (≥3 months old) male and female transgenic mice in which green fluorescent protein (GFP) is expressed under the chemokine receptor promoter, Cx3cr1, were used for microglia labeling. Cx3cr1^*GFP*^ animals (RRID:IMSR_JAX:005582) carry an enhanced green fluorescent protein (GFP) gene “knocked in” to replace the first 390 base pairs of the second exon region of the fractalkine receptor gene^85^. These animals were used for two-photon analyses.

For histological analyses and confocal microscopy, we combine this model with *Ccr2*^*RFP*^ animals (RRID:IMSR_JAX:017586) to genetically label infiltrating peripheral myeloid cells^30^. In these animals, red fluorescent protein (RFP) replaces the first 279 base pairs of the CCR2 open reading frame^86^. This model labels peripheral monocytes, T-cells, dendritic cells, and other small populations of myeloid-derived cells. Experimental mice were produced by crossing *Cx3cr1*^*GFP/GFP*^ with *Ccr2*^*RFP/RFP*^ mice to generate dual heterozygous *Cx3cr1*^*GFP/+*^*::Ccr2*^*RFP/+*^mice. The animals were kept in a C57Blk/6 J mixed background.

### Motoneuron pre-labeling

Motoneurons innervating the lateral gastrocnemius (LG) muscle were pre-labeled by injecting 5 µl of 0.1% cholera toxin subunit b conjugated to an Alexa Fluor 555 (CTb-555) (Invitrogen) or 1.5% of Fast Blue (Polysciences) directly into the LG muscle with a Hamilton syringe. Buprenorphine (0.05–0.10 mg/kg) was injected subcutaneously (s.c.) for postsurgical pain management. Retrograde labeling was done one week prior to any nerve injury and under isoflurane anesthesia (induction: 4–5%; maintenance: 2–3%, both in 100% O_2_). CTb-555 animals were used for dual color two-photon imaging with CX3CR1-GFP microglia. Fast Blue animals were used for histological processing or to confirm the location of axotomized motoneurons with epi-fluorescence prior to two photon imaging of CX3CR1-GFP microglia.

### Nerve Injury

Mice were anesthetized with isoflurane until a surgical plane of anesthesia was achieved (induced: 4–5%; maintained: 2–3%, both in 100% O_2_) and treated with s.c. buprenorphine as above. Once animals were anesthetized, a posterior hindlimb incision was made on the left side. The biceps femoris muscle was blunt dissected and the sciatic nerve exposed, dissected free of connective tissue, and completely transected with surgical scissors. A small piece of 5/0 sterile silk was tied around the proximal nerve stump to prevent regeneration from taking place. Both the dissected muscle and skin were sewn shut with absorbable suture. During the first week post-operation, all animals were monitored daily for surgical complications or signs of distress. Mice were allowed to survive for 3 to 14 days post-injury.

### Slice Preparation

We prepared “dissection” artificial cerebrospinal fluid (DaCSF) and “imaging” aCSF (IaCSF) (Table 1) optimized to preserve the health and integrity of adult spinal cord slices^50^. Dissection aCSF had all Na^+^ ions removed to prevent neuronal firing and substituted for isomolar concentrations of sucrose. This modification aids in motoneuron preservation in adult slices^87^. In addition, calcium was reduced and magnesium increased to dampen synaptic activity and NMDA-receptor activation. Osmolarity of both aCSF solutions ranged between 305–315 mOsm/kg of H_2_O, pH was between 7.3–7.4 and both solutions were oxygenated with 95% O_2_/5% CO_2_ for 30 minutes prior to use.

**Table 1:** Composition of aCSF solutions.

Dissecting aCSF (DaCSF)		Imaging aCSF (IaCSF)	
Concentration (mM)	Compound	Concentration (mM)	Compound
191	Sucrose	121	NaCl
0.75	K-gluconate	3	KCl
1.25	KH_2_PO_4_	1.25	NaH_2_PO_4_
26	Choline bicarbonate (80%)	25	NaHCO_3_
4	MgSO_4_	1.1	MgCl_2_
1	CaCl_2_	2.2	CaCl_2_
20	Dextrose	15	Dextrose
1	(+) sodium L-ascorbate	1	(+) sodium L-ascorbate
5	Ethyl pyruvate	5	Ethyl pyruvate
3	Myo-inositol	3	Myo-inositol
2	Kynurenic acid sodium salt		

#### Spinal cord dissection

Mice were injected with ≥100 mg/kg of Euthasol until a deep plane of anesthesia was reached. At this point the mice were quickly decapitated and the spinal cord rapidly removed (about 2–3 mins) under a dissection microscope using a petri dish with a Sylgard base filled with oxygenating DaCSF maintained at low temperature (0–4 °C) by an underlying Peltier plate. The meninges were peeled off with #5 forceps and the lumbar region cut and transferred to pre-warmed (35 °C) and oxygenating DaCSF where they were incubated for 15 minutes at room temperature.

#### Sectioning

We followed the methodology from Mitra and Brownstone^50^. While the tissue incubates, we prepared fresh low-melting 4% agarose (Sigma) mixed in oxygenated DaCSF on a heat stirring plate and cooled to approximately 37 °C. During the last minute of tissue incubation in DaCSF, some of the agar was poured in a 15 mm petri dish placed on ice, the tissue was then added and covered with additional agar and the petri dish quickly placed in a −20 °C freezer for about 5 mins to accelerate agar solidification. Once the agar solidified, the spinal cord was blocked out with a razor blade, leaving only a small layer of agar on all sides of the cord. The agar block was secured vertically to the cutting tray of a vibratome (Leica VT1000P) and glued with “superglue”. To obtain almost instantaneous glue hardening we sprinkled bicarbonate power which was then quickly washed with DaCSF before filling the reservoir. The cutting tray was then filled with ice cold oxygenated DaCSF, and 350 µm thick transverse sections cut and collected by suction using a cut and fire-polished glass transfer pipette with an approximately 5 mm opening. The sections were then placed in 30% polyethylene glycol (PEG, pre-heated to 35 °C, mixed in dH_2_O and oxygenated) for 1 to 2 mins. This step speeds the sealing of cut cell processes in the low calcium conditions of the DaCSF^88^. After several washes with 35 °C DaCSF, the sections were transferred to a 35 °C incubation chamber filled with DaCSF and constant oxygenation for 30 minutes. Finally, the sections were transferred to a chamber filled with IaCSF at room temperature and constant oxygenation for another 30 minutes before imaging.

### Spinal cord slice validation and immunocytochemistry

To test whether microglia are rapidly activated by the sectioning process, we prepared a series of spinal cord slices as above and after a waiting period of 1, 2, 3, 4, 5, or 6 hours after slicing, they were fixed for 1 hour with 4% paraformaldehyde in 0.1 M phosphate buffer (PB, pH 7.4). After thorough washing, the slices were blocked in donkey normal serum diluted 1:10 in 0.01 phosphate buffer saline, pH 7.2–7.3 with 0.3% Triton-X-100 (PBS-TX) and placed overnight in a rat monoclonal antibody (FA-11) against CD68 (AbCam ab53444, RRID:AB_869007) diluted 1:100 in PBS-TX. Immunoreactive sites were revealed the following day using donkey anti-rat IgG antibodies coupled to Cy3 (Jackson ImmunoResearch Labs, West Grove, PA). Finally, sections were washed, mounted and coverslipped with Vectashield (Vector Labs, Burlingame, CA) and imaged in a FV1000 Olympus confocal microscope.

### Analysis of CD68 and microglia morphology in histological sections

CD68 expression and cell morphology were also analyzed in microglia ipsilateral and contralateral to the injury at different post-injury times and from animals perfusion-fixed with 4% paraformaldehyde and processed for routine histological immunolabeling. In this case, LG motoneurons were retrogradely labeled with Fast Blue (Polysciences) and the nerve injury performed 1 week after. At 3, 7 and 14 days after the nerve injury, the animals were deeply anesthetized with Euthasol (100 mg/kg) and transcardially perfused first with vascular rinse containing heparin followed by 4% paraformaldehyde in 0.1 M phosphate buffer (PB, pH 7.4). The spinal cords were post-fixed overnight in the same fixative solution at 4 °C. The following day they were cryoprotected in 30% sucrose at 4 °C. Transverse 50 µm thick sections were obtained on a freezing sliding microtome and processed free floating. We combined a chicken polyclonal antibody against GFP (Serotect, #obt1644, RRID:AB_620519) with the FA-11 monoclonal antibody (anti-CD68). Primary antibody incubations were done overnight at room temperature. GFP immunoreactivity was revealed with FITC-conjugated anti-chicken IgY antibodies and FA-11 reactivity with Cy3 conjugated anti-rat IgG antibodies. Both secondary antibodies were generated in donkey (Jackson ImmunoResearch Labs).

### Analysis of microglia, CD68 and synaptic markers in histological sections

In 50 µm thick frozen sections from the spinal cord of paraformaldehyde-perfused animals 14 days after sciatic nerve injury and retrograde transport of Fast Blue in LG motoneurons we combined FA-11 CD68 immnuoreactivity with antibodies against Iba1 (Goat/polyclonal, diluted 1:500; Wako #019–19741 RRID:AB_839504), VGLUT1 (Rabbit/polyclonal, diluted 1:500; Synaptic Systems #135303 RRID:AB887875), VGLUT2 (Rabbit/polyclonal, diluted 1:500; Synaptic Systems #135402 RRID:AB887883) or VGAT (Mouse/monoclonal, diluted 1:500; Synaptic Systems #131011 RRID:AB887872). In this case, Iba1+ microglia was revealed with FITC-conjugated antibodies, synaptic markers with Cy3-conjugated anti-rabbit or anti-mouse IgG antibodies and FA-11 immunoreactivity with Cy5 or DyLight 647 conjugated anti-rat IgG antibodies.

#### Antibody specificity

The FA-11 monoclonal antibody recognizes murine macrosialin, a heavily glycosylated transmembrane protein homolog to human CD68. This murine macrophage glycoprotein antigen is intracellularly expressed in lysosomes and it has been widely used as a pan macrophage marker since it was first discovered after the creation of the FA-11 antibody^89^. The antibody was originally generated using as immunogen a preparation of concanavalin A acceptor glycoproteins isolated from P815 cells. The anti-GFP antibody did not recognize any background labeling in the absence of GFP expression (i.e. sections from wild-type animals). Iba1, VGLUT1, VGLUT2 and VGAT antibodies showed immunoreactivity patterns and laminar densities identical to those previously shown to be specific for each of these antigens in the spinal cord, and sometimes confirmed in KO tissues^30,90,91^.

### Two-photon imaging

Sections were secured in a slice imaging chamber with a harp to prevent movement and placed on top of a stage platform under a HC-Fluotar-L 25x water immersion objective (NA = 0.95). A circulating bath of IaCSF delivered oxygenated solution to the sections in between imaging sets. We stopped the flow during imaging to prevent movement artifacts due to fluid flow. Time-lapse video data sets were captured using a Leica SP8 two-photon microscope equipped with a Coherent Chameleon Vision II laser tuned to 920 nm and at 2–5% strength. EGFP and CTb-555 fluorescence were simultaneously captured with high sensitive external non-descanned gallium-arsenide HyD detectors (BrightR mode). Each fluorochrome emission was separated into a green or red channel using standard filter settings. Maximization of Alexa-555 sensitivity provoked extensive bleed of EGFP fluorescence into the red channel, while none of the red fluorescence entered the green channel. We used two different approaches to overcome this problem. First, both channels were separated using the Leica’s linear un-mixing algorithm. Second, when working in Imaris, we transformed the green channel into white pseudo color to fully distinguish it from red only. Digital zoom was set up at different levels depending on the size of the field of view necessary to include cells targeted for imaging. We set up z-stacks to a 1.5 µm step size to scan volumes of approximately 45 µm average thickness. Scan speed (laser dwell time per pixel) was set at 400 Hz and images were 512 × 512×/y pixel size. In these imaging conditions each volume stack was captured approximately every 30 seconds to 1 minute (depending on total volume thickness) with little or no dwell time between scans. Total recording times lasted 30–60 mins. Longer time-lapses (> 90 minutes) frequently resulted in altering microglia morphology and activity, likely due to changes in bath temperature during continuous infrared illumination. All analyses are from microglia recorded for less than 60 minutes, and this was considered a minor problem compared to using a bath flow for efficient temperature exchange and cooling.

### Analyses

#### Microglia surface, volume and CD68 content

High magnification (60×) confocal images were obtained of microglia taken from spinal cord slices at different times post-slicing (1–6 hours) or from histological sections of perfusion fixed animals. Data sets were uploaded to Imaris (Bitplane, version 7.2.2) and 3D renderings created using the surface area module for both microglia surface (CX3CR1-GFP) and CD68 immunoreactivity. We compared surface area of microglia, cell body volume and the volume occupancy of CD68 at different time points and conditions. Six microglia were analyzed per time point and preparation. Each preparation was obtained from one animal.

#### Microglia coverage of motoneuron surface

High magnification (60×) confocal images were obtained from histological sections containing Fast Blue motoneurons and CX3CR1-GFP microglia 3, 7 and 14 days after nerve injury. We randomly selected 10–12 motoneurons from controls and at each post-injury date from a total of two animals per time point. We selected motoneurons with the cell body contained within the optical sections stack. The cell body surface area was reconstructed using the “surface module” in Imaris software. We then obtained surface reconstructions of all microglia touching the motoneuron. The “surface-surface contact area XTension” MATLAB plug-in provided by Bitplane was used to compute the total surface of microglia in direct contact with the motoneuron cell body surface to estimate the percent of motoneuron cell body surface covered by microglia.

#### Changes in microglia surface after activation

Data for these analyses was collected from 3 mice euthanized 3 days post-injury. For each animal, we recorded 4–6 videos/files, each lasting 30–60 minutes. Data was pulled from a mix of individual video files which were selected based on the stability of the recording session (i.e., minimal slice and stage drift, and lowest amount of noise). Time-lapse data sets were analyzed with Imaris. The surface of microglia was reconstructed and calculated at each time point during the first 30 minutes of time lapse imaging and the percentage change calculated. We analyzed 6 microglia contralateral to the nerve injury (controls) and 6 ipsilateral to the nerve injury.

#### Phagocytic cup analysis

The same time-lapse data sets were opened in ImageJ and the number of phagocytic cups formed over a 30 minutes imaging period counted and compared between control and activated microglia 3 days post-nerve injury. The duration of each cup was quantified from the initiation of the extension of the parent branch to its return. We analyzed the same 6 microglia contralateral and 6 ipsilateral to the nerve injury as in the analysis of surface changes.

#### Filament tracing of microglia process dynamics

Time-lapse data sets were uploaded to Imaris and individual filaments that extend off the cell body were reconstructed in 4D using the manual function of the filament tracer module. We then quantified the total process change in length over a 30-minute imaging session. The terminal-end motility was calculated by the change in length of a terminal process from the last branch point to the end of the process. We analyzed the same sample of microglia used for changes in surface area with time (6 control, 6 activated).

#### CCR2 cell infiltration and CD3/CD8 analysis

We performed sciatic nerve injuries in 4 dual heterozygous *Cx3cr1*^*GFP/*+^*::Ccr2*^*RFP/*+^ mice to analyzed CX3CR1 and CCR2 cells in relation to axotomized Fast Blue motoneurons 14 days after nerve injury. In addition, we used immunohistochemistry, as described above, to label T-cells with a mouse monoclonal antibody against CD3 (Clone 500A2 25 kDa e chain; BD Biosciences #553238 RRID:Ab_394727) and cytotoxic T-cells with a rabbit polyclonal antibody against CD8 (Abcam ab4055 RRID:Ab_304247). GFP was detected as above.

### Statistics

We recorded multiple cells per imaging session (1 imaging session = 1 animal), therefore statistics are based on pooling cells from different experiments and grouping them by condition (i.e., time after injury or time after slicing; control side vs injured side). For multiple comparisons (i.e., time after slicing), we used one-way ANOVAs followed by Bonferroni post-hoc t-tests for pair-wise comparisons. When normality failed, we used a non-parametric Kruskal-Wallis one-way ANOVA on ranks followed by a Dunn’s Method post-hoc tests. For two sample comparisons (i.e., microglia in injured or non-injured side), we used two-tailed t-tests. Specific statistical comparisons in each experiment are indicated in the text and figure legends. All alpha values were set at 0.05. Power was always>0.800, unless indicated. A mix of male and female mice were used. We detected no sex-specific trends and thus, male and female data were pooled together.

## Supplementary information

Supplementary movie information.

Supplementary Movie 1

Supplementary Movie 2

Supplementary Movie 3

Supplementary Movie 4

Supplementary Movie 5

Supplementary Movie 6

Supplementary Movie 7

Supplementary Movie 8.

Supplementary Movie 9.

Supplementary Movie 10

Supplementary Movie 11

Supplementary Movie 12

Supplementary Movie 13

Supplementary Movie 14
