# Supplementary material for: Microglia Dynamics and Interactions with Motoneurons Axotomized After Nerve Injuries Revealed By Two-Photon Imaging
Source: Sci Rep. 2020 May 26;10:8648. doi: 10.1038/s41598-020-65363-9 (PMC7250868; doi:10.1038/s41598-020-65363-9)
Supplement: Supplementary file 1 — Supplementary movie information. [file 41598_2020_65363_MOESM1_ESM.pdf]

MICROGLIA DYNAMICS AND INTERACTIONS WITH MOTONEURONS AXOTOMIZED AFTER NERVE INJURIES REVEALED BY TWO-PHOTON IMAGING

Travis M. Rotterman[1,2] & Francisco J. Alvarez[1]*

[1]Department of Physiology, Emory University, Atlanta, GA 30322

[2]School of Biological Sciences, Georgia Tech, Atlanta, GA 30318

Supplementary Movies

Supplementary movie 1. Low magnification of activated microglia in the ventral horn of the spinal cord ipsilateral to sciatic nerve injury. Relates to Figure 3b

Total time, 21 mins 29sec; Each frame captured every 30.4 secs (42 frames); Playback 5 fps.

Supplementary movie 2. Dynamics of control microglia. Relates to Figure 3c

Total time, 45 mins 53sec; Each frame captured every 1 minute and 2.6 secs (43 frames); Playback 5 fps.

Microglai showing process expanding and retracting at normal rates and with many filopodia.

Supplementary movie 3. Dynamics of activated microglia. Relates to Figure 3c

Total time, 44 mins 11sec; Each frame captured every 1 minute and 0.2 secs (43 frames); Playback 5 fps.

Three microglia are shown. The cell at the top contains many large phagosomes and very active formation of phagocytic cups stemming almost directly from the cell body. The cells below show longer processes and phagocytic cup formation at the end of processes.

Supplementary movie 4. Phagosome traveling. Relates to Figure 3h

Total time, 26 mins; Each frame captured every 35 secs; Playback 5 fps.

Phagosomes are seen traveling along microglia processes and disappearing upon reaching the cell body.

Supplementary movie 5. Filament tracking of microglia processes. Relates to Figure 41

Total time, 40 minutes; Each frame captured every 35 secs; Playback 10 fps.

Dots represent microglia process endings. Filaments indicate best fit trajectories through processed toward the cell body.

Supplementary movie 6. Interactions between surveying non-activated microglia and non-injured lateral gastrocnemius motoneurons (labeled from muscles with CTb-555).

Relates to Figure 5a

Total time, 5 mins; Each frame captured every 32.19 secs; Playback 5 fps.

Microglia extend process towards the motoneuron processes that after a brief scan are quickly retracted.

S**upplementary movie 7. Interactions between activated microglia and lateral gastrocnemius motoneurons (labeled from muscles with CTb-555) 7 days after axotomy.**

Relates to Figure 5b

Total time, 14mins 50s; Each frame captured every 30 secs; Playback 5 fps.

Microglai extend processes that end in phagocytic cups at the motoneuron surface. Some microglia processes however remain attached for long period of times extending and retracting filopodia

Supplementary movie 8. Interactions between activated microglia and lateral gastrocnemius motoneurons (labeled from muscles with CTb-555) 10 days after axotomy.

Relates to Figure 5c

Total time, 13mins 30s; Each frame captured every 30 secs; Playback 5 fps.

Microglia are stationary on te surface of axotomized motoneurson extending numerous filopodia.

Supplementary movie 9. Imaris 3D analysis of microglia-motoneuron surface coverage over a control motoneuron.

Relates to Figure 6a-c

Blue represents a Fast Blue retrogradely labeled lateral gastrocnemius motoneuron, green microglia surface and red areas of apposition between motoneuron and microglia surfaces.

Supplementary movie 10. Imaris 3D analysis of microglia-motoneuron surface coverage in over a motoneuron 14 days after axotomy.

Relates to Figure 3d

Blue represents a Fast Blue retrogradely labeled lateral gastrocnemius motoneuron, green microglia surfaces and red areas of apposition between motoneuron and microglia surfaces.

Supplementary Movie 11. Confocal image stacks showing accumulation of single optical planes with 0,5 µm Z-steps and showing lack of colocalization between CD68 granules and VGLUT2 and no VGLUT2 synapses incorporated within microglia.

Relates to figure 7b1

Blue represents two Fast Blue retrogradely labeled lateral gastrocnemius motoneurons; green, microglia surfaces; white, CD68 phagosomes; red, VGLUT2 synaptic boutons.

Supplementary Movie 12. High magnification Imaris 3D volume rendering showing a lack of VGLUT2 boutons incorporation in either CD68 granules or the microglia volume.

Relates to figure 7b2

Blue represents two Fast Blue retrogradely labeled lateral gastrocnemius motoneurons; green, microglia surfaces; white, CD68 phagosomes; red, VGLUT2 synaptic boutons.

Supplementary Movie 13. Confocal image stacks showing accumulation of single optical planes with 0,5 µm Z-steps and showing lack of colocalization between CD68 granules and VGLUT1 and no VGLUT1 synapses incorporated within microglia.

Relates to figure 7c1

Blue represents two Fast Blue retrogradely labeled lateral gastrocnemius motoneurons; green, microglia surfaces; white, CD68 phagosomes; red, VGLUT1 synaptic boutons.

Supplementary Movie 14. High magnification Imaris 3D volume rendering showing a lack of VGLUT1 boutons incorporation in either CD68 granules or the microglia volume.

Relates to figure 7c2

Blue represents two Fast Blue retrogradely labeled lateral gastrocnemius motoneurons; green, microglia surfaces; white, CD68 phagosomes; red, VGLUT2 synaptic boutons.
